# Case report: Acne vulgaris treatment with 5-Aminolaevulinic acid photodynamic therapy and adalimumab: a novel approach

**DOI:** 10.3389/fmed.2023.1187186

**Published:** 2023-05-12

**Authors:** Yang Ping, Zhong Jian Bo, Zhao Xing Yun, Kamran Ali, Chen Jun, Inmaculada Xu Lou, Li Ming Wu

**Affiliations:** ^1^Department of Dermatology, Affiliated Hangzhou First People's Hospital, Zhejiang University School of Medicine, Hangzhou, Zhejiang, China; ^2^Department of Oncology, The Fourth Affiliated Hospital, International Institutes of Medicine, Zhejiang University School of Medicine, Yiwu, China; ^3^Department of Cardiology, International Education College of Zhejiang Chinese Medical University, Hangzhou, Zhejiang, China

**Keywords:** adalimumab, 5-Aminolaevulinic acid (ALA) photodynamic therapy (PDT), acne vulgaris, TNF inhibitors, biological agent

## Abstract

Acne vulgaris is a common skin condition that affects a large proportion of teenagers and young adults. Despite the availability of various treatment options, many patients experience inadequate relief or intolerable side effects. Photodynamic therapy (PDT) is a growing interest in the treatment of acne vulgaris, with 5-Aminolaevulinic acid (ALA) being one of the most commonly used photosensitizers. Adalimumab is a biologic medication used to treat inflammatory skin conditions such as Psoriasis and Hidradenitis suppurativa (HS), which targets TNF-α. Combining different therapies, such as ALA-PDT and adalimumab, can often provide more effective and longer-lasting results. This report presents the case of a patient with severe and refractory acne vulgaris who was treated with a combination of ALA-PDT and adalimumab, resulting in significant improvement in the condition. The literature review highlights the significant comorbidity associated with acne, emphasizing the need for potential of TNF-α inhibitors for its effective treatments that address physical symptoms and ALA-PDT is known to treat scar hyperplasia, and to prevent or minimize the formation of post-acne hypertrophic scars. The combination of TNF inhibitors and ALA-PDT or adalimumab has shown promising results in treating inflammatory skin conditions, including severe and refractory acne vulgaris, as per recent studies.

## 1. Introduction

Acne vulgaris, a chronic inflammatory skin condition, is a prevalent dermatological disorder that primarily affects teenagers and young adults, with an estimated incidence of ~85% (1). It is characterized by the formation of comedones, papules, pustules, and nodules on the face, neck, chest, and back due to the obstruction of hair follicles by excess sebum and keratin, leading to the proliferation of *Propionibacterium acnes* and subsequent inflammation (2–4). Despite the availability of numerous treatment options, including topical and oral medications, many patients experience inadequate relief or intolerable side effects (5). Long-term treatment of acne, the constant appearance of skin eruptions, and acne scars can negatively affect mental and physical health. The presence of this disease causes discomfort that can lead to emotional disorders, reduced quality of life, and depression (6). The growing interest in the use of photodynamic therapy (PDT) for treating acne vulgaris has led to the investigation of various photosensitizers. One of the most commonly used agents in PDT is 5-Aminolaevulinic acid (ALA). ALA-PDT involves the topical application of ALA, which is then activated by a specific wavelength of light to destroy targeted cells. In addition to PDT, biologic medications that target specific inflammatory mediators have also been developed for the treatment of skin conditions. Adalimumab, a biologic medication that targets tumor necrosis factor alpha (TNF-α), is effective in treating inflammatory skin conditions such as psoriasis and Hidradenitis suppurativa (HS) (7, 8). While various treatment options are available, combining different therapies can often provide more effective and longer-lasting results. The concurrent utilization of 5-Aminolaevulinic acid (ALA) photodynamic therapy (PDT) and adalimumab represents a potential therapeutic combination. In this report, we describe the case of a patient who presented with severe and refractory acne vulgaris. Despite multiple treatment attempts with various topical and systemic agents, the patient's condition remained resistant to therapy. Therefore, a combination of aminolevulinic acid photodynamic therapy (ALA-PDT) and adalimumab (a monoclonal antibody targeting TNF-α) was initiated.

## 2. Case presentation

An 18-year-old male patient presented with recurrent papules and nodules on the face persisting for a duration of 2 years. The patient had severe acne and had been treated with doxycycline 100 mg twice a day orally then isotretinoin 20 mg per day, and topical (Fusidic acid twice a day, and Tretinoin once a day) and, along with fire acupuncture treatment. Four weeks after treatment, the patient was administrated prednisone tablets 25 mg per day for a week and isotretinoin 20 mg per day which improved the condition. The prednisone dose was gradually reduced to 10 mg/day for a week and isotretinoin 20 mg/day, but the condition suddenly aggravated. The prednisone dose was increased to 25 mg/day for a week and isotretinoin 20 mg/per day was again administered, but the condition continued to aggravate.

**Figure 1:**
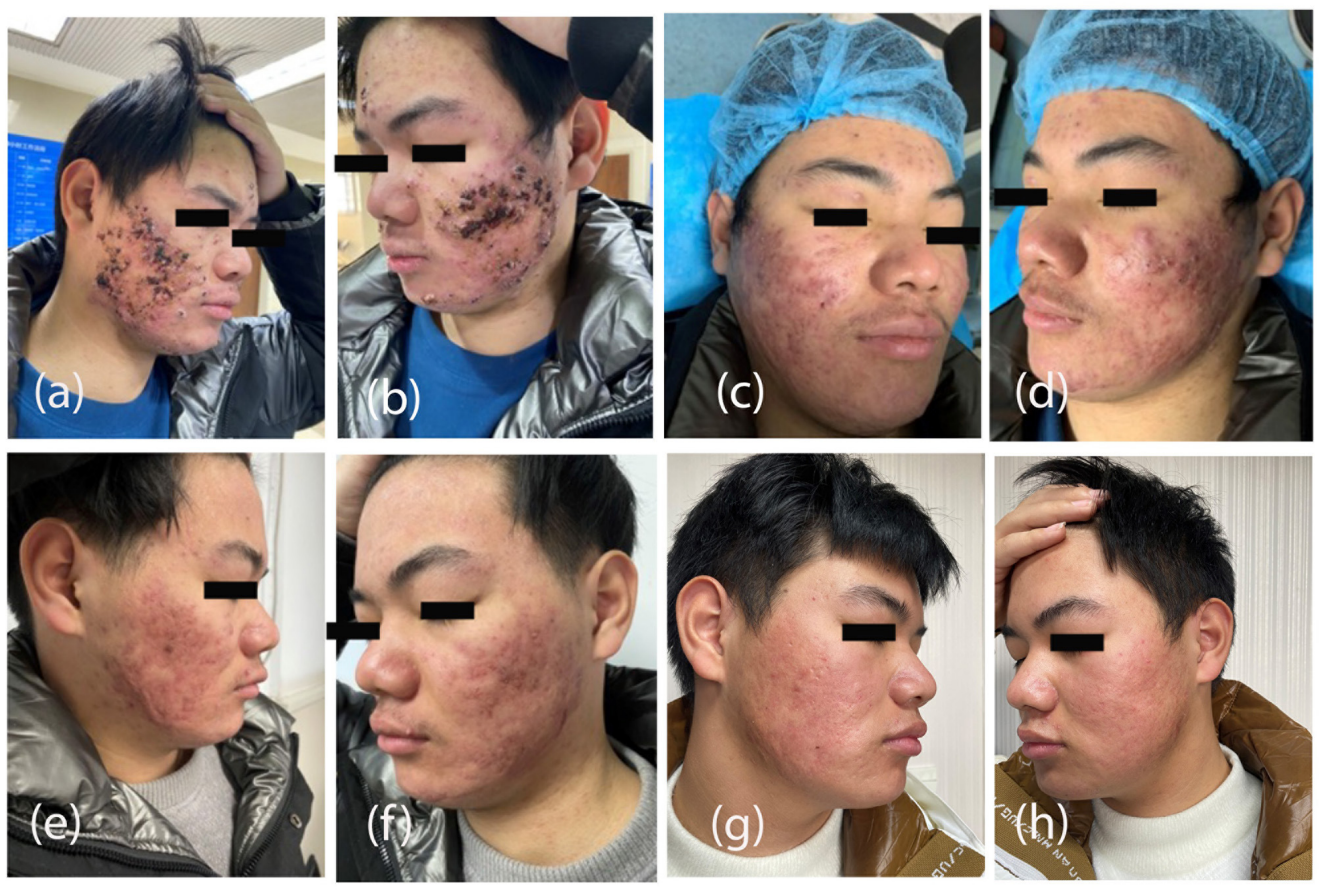
Progression of treatment for acne vulgaris (swollen abscess and nodule) using adalimumab and photodynamic therapy. At the time of admission at the hospital, the image shows a swollen and inflamed abscess with a nodule **(a, b)**. On 1st follow-up of adalimumab + photodynamic therapy, the abscess subsided significantly, and the nodule became softer and narrower than before **(c, d)**. After the 2nd treatment of adalimumab and photodynamic therapy, the abscess and nodules have significantly subsided, leaving scattered papules and dark red scars **(e, f)**. After four months of maintenance therapy (isotretinoin), the lesions did not recur even after discontinuation of the drug **(g, h)**.

The patient had repeated papules and nodules on the face (Figures 1a, b). The patient was hospitalized, after admission, prednisone treatment was stopped, and ALA photodynamic therapy was initiated once a week for 3 consecutive weeks. Adalimumab (Humira) 40 mg subcutaneously was injected once every 2 weeks for three consecutive times, and the rash gradually improved. Before the first photodynamic therapy, the pretreatment scab was removed, the abscess was opened with ionization, pus was squeezed out, and then the drug was applied with a 10% concentration of 5-ALA. Fusidic acid twice a day was continued to use during combination therapy and during follow-up period.

After one session of adalimumab + photodynamic therapy, the abscess subsided significantly, and the nodules became softer and smaller than before (Figures 1c, d). After two treatments with adalimumab + photodynamic therapy, the abscess and nodules basically subsided, with scattered papules and dark red scars. The follow-up visit showed that the nodules and papules had disappeared, leaving dark red scars, and there was no new rash (Figures 1e, f).

The patient had severe acne and tended to have congregated acne, and traditional treatment was ineffective. After one session of photodynamic therapy alone, the condition improved slightly, but the effect was not satisfactory. The symptoms improved rapidly after adalimumab (Humira) + photodynamic combined therapy. It has proven that adalimumab treatment helps control the disease to be controlled rapidly and reduces hypertrophic scar formation in later stages. Early treatment can reduce the formation of post-acne hypertrophic scars. After the short-term combined treatment, the patient continued to take traditional treatment (orally isotretinoin) for 4 months, and the skin lesions did not recur after stopping the drug (Figures 1g, h). There was no recurrence of the rash observed after 1 year follow-up.

## 3. Literature review

### 3.1. Acne vulgaris

Acne vulgaris, a prevalent inflammatory disorder of the pilosebaceous unit of the skin, predominantly affects the face and trunk. It is estimated to impact about 9% of the global population, with a higher prevalence among individuals aged 12–24 years (about 85%) and a considerable percentage of patients aged 20–29 years (around 50%) (9). In addition to its physical symptoms, acne is also known to cause scarring in affected areas. These scars have significant psychological and social implications, including an increased risk of suicide, depression, poor academic performance, unemployment, and poor quality of life for those afflicted with this condition (10). Therefore, acne scars represent a significant comorbidity associated with acne, emphasizing the need for effective treatments that not only address physical symptoms but also prevent or minimize scarring (11, 12). Acne is characterized by the elevation of certain cytokines, including TNF-α, IL-1β, and granulocyte-macrophage colony-stimulating factor. Although the exact mechanism and cause of this inflammation are still unknown, research suggests that it may be related to *Propionibacterium acnes*. Therefore, recent studies support the use of TNF inhibitors in the treatment of this condition (13).

### 3.2. Aminolaevulinic acid photodynamic therapy for acne vulgaris

5-Aminolevulinic acid-based photodynamic therapy (ALA-PDT) is a novel and effective approach for the treatment of severe acne vulgaris (14, 15). The most widely used photosensitizers are 5-Aminolevulinic acid and methyl aminolevulinate, both of which have comparable clinical efficacy (14). The mechanism of PDT action is considered to reduce sebum excretion and the colonization of Cutibacterium acnes, while its immunomodulatory effects have also been described (15). However, the specific treatment mechanism of ALA-PDT for acne vulgaris still remains unclear (15).

ALA-PDT is a relatively new therapeutic technique that is used widely in dermatology (16). It is a clinical procedure that involves the application of a photosensitizing agent, such as ALA, to the skin, followed by exposure to a light source (17). The photosensitizing agent is absorbed by the skin and converted to a substance that is activated by light. When the light is applied, the activated substance produces a reaction that destroys the acne-causing bacteria.

Several clinical guidelines for acne recommend PDT as an alternative treatment modality for severe acne (17, 18). Studies have shown that ALA-PDT is clinically effective in the management of acne vulgaris (18, 19). A study of clinical efficacy and mechanism of action found that topical ALA-PDT was effective in reducing the number of inflammatory acne lesions (20). Another study found that ALA-PDT amplified an intense inflammatory response in the treatment of acne vulgaris via CXCL8 (15). ALA-PDT has also been found to be effective in reducing the number of non-inflammatory acne lesions (16).

In a study investigating the potential treatment mechanism of ALA-PDT for acne vulgaris, it was found that ALA-PDT amplifies intense inflammatory response in the treatment of acne vulgaris via CXCL8 (15). Another study found that ALA can penetrate the skin and convert to protoporphyrin IX (PpIX) to exert the photodynamic response (16). Topical ALA-PDT for the treatment of acne vulgaris has been found to be clinically effective and has been recommended as an alternative treatment modality for severe acne (14, 18).

The mechanism of action of ALA-PDT for acne vulgaris is not fully understood, but it is believed to reduce sebum excretion and the colonization of Cutibacterium acnes, while its immunomodulatory effects have also been described (21). ALA-PDT has been found to be clinically effective and has been recommended as an alternative treatment modality for severe acne.

### 3.3. Aminolaevulinic acid photodynamic therapy and adalimumab

Aminolevulinic acid (ALA) is a photosensitizing substance used in photodynamic therapy (PDT) for treating various medical conditions, such as certain types of cancer and skin diseases. PDT involves the administration of ALA, which selectively accumulates in diseased cells, followed by exposure of the affected area to visible light or laser. The interaction of light with ALA causes the release of reactive oxygen species that damage and destroy diseased cells. PDT is a non-invasive and effective treatment option for many diseases and offers an alternative to surgery and radiation (15, 22). The efficacy of 5-Aminolaevulinic acid-mediated photodynamic therapy (ALA-PDT) for the treatment of acne vulgaris has been extensively investigated in numerous studies. Clinical trials have provided evidence for the significant reduction of both inflammatory and non-inflammatory acne lesions following ALA-PDT (23, 24). Therefore, ALA-PDT can be considered as an alternative therapeutic option for severe cases of acne vulgaris when conventional treatment modalities fail to deliver the desired outcomes (25, 26). However, its main limitation is that it requires multiple sessions and may increase the risk of photosensitivity (27). On the other hand, Adalimumab (ADA) is a monoclonal antibody that inhibits TNF-α and has been approved in Europe, Australia, and North America for the treatment of various inflammatory disorders, including Crohn's disease, psoriasis, rheumatoid arthritis, ankylosing spondylitis, and ulcerative colitis. However, relatively little is known about its efficacy and safety in the treatment of acne vulgaris (13, 28–30).

### 3.4. Adalimumab for Hidradenitis suppurativa/acne inversa

Hidradenitis suppurativa (HS), commonly known as acne inversa, is a chronic and recurrent inflammatory skin disease that predominantly affects adolescents and is often debilitating. This dermatological condition is characterized by the presence of inflamed and painful lesions in the regions with apocrine glands, such as the anogenital region, armpits, and inguinal area (28). In the general population, there is a strong association with obesity and smoking (31). Among pediatric patients, this disease is highly associated with other skin comorbidities and medical conditions such as acne vulgaris, acne conglobate, obesity, and anxiety (32). ADA is the first drug approved for the treatment of moderately to severely active HS in patients who have not responded to conventional systemic therapies (33). The standard dosage of 40 mg per week of ADA has been shown to be both effective and safe for the long-term treatment of moderate-to-severe HS, as evidenced by improvements in lesion counts, skin pain, and Dermatology Life Quality Index (DLQI) (28, 34).

Hidradenitis suppurativa (HS) is a multifactorial pathology with occlusion of the hair follicle leading to perifollicular lymphohistiocytic infiltration as the main cause. TNF-α levels are generally elevated in both blood and injured and perilesional skin. ADA treatment has been shown to decrease the production of cytokines, such as IL-1β, CXCL9, and BLC, and inflammatory cells such as CD11c+, CD14+, CD68+, and CD3+ and CD4+ T cells. Active inflammatory lesions in moderate-to-severe HS typically improve after 12 weeks of ADA treatment. However, ADA is ineffective against non-inflammatory scarring lesions, which require a combined treatment approach involving radical surgery (31, 35).

### 3.5. Adalimumab for acne fulminans

Acne fulminans (AF) is a highly inflammatory form of acne. In the scientific literature, there are fewer than 200 documented cases, making it a rare disease. Similar to HS, it is also difficult to treat. AF is seen more frequently in adolescent males between the ages of 13 and 22 years, with a previous history of acne vulgaris (36). It is postulated that the main triggering agents are due to an explosive hypersensitivity reaction to the bacterium *Propionobacterium acnes*, as well as an increase in androgens (37). Although isotretinoin and steroids are the first-line treatments for acne, some patients may exhibit resistance to these drugs, and in some cases, their use may exacerbate the condition (36). In cases where other treatments have proven ineffective administration of subcutaneous ADA at a dose of 80 mg followed by 40 mg every 2 weeks for a period of 3–4 months can effectively improve lesions and inflammatory scars, without the risk of remission (38). Kontochristopoulos et al. (29) reported significant improvement in a patient with HS and AF who did not respond to conventional treatment with clindamycin and rifampicin following the administration of ADA (29). The study conducted by Rajaii et al. (37) administered subcutaneous ADA at a dose of 40 mg every 2 weeks, in combination with oral doxycycline, after discontinuing treatment with clindamycin, tretinoin, and steroids for 12 months due to a lack of clinical improvement. Following 6 weeks of treatment with a TNF-α inhibiting drug, a noteworthy reduction in inflammatory nodules, cysts, papules, pustules, and abscesses was observed (37). Depression is a common problem among patients with acne. In some cases, medications, such as retinoids, cannot be prescribed because of their potential to cause headaches and exacerbate this psychological illness. In such scenarios, adalimumab (ADA) can be administered at a standard dose of 40 mg/week. This was demonstrated in a study by Miguel et al. (39), who combined ADA with 20 mg/day of prednisolone and 100 mg/day of minocycline. The inflammatory lesions in the patient reported in this case resolved, and the response was sustained for at least 6 months of follow-up (39).

### 3.6. Adalimumab for acne conglobata

Acne conglobate is a variant of acne, that manifests as inflammation and it is severe and chronic. It presents as cystic nodules, abscesses, and sinus tracts. Conventional acne treatments are often ineffective in the case of acne conglobate. However, ADA at a dose of 40 mg/week has also been shown to be effective and safe in patients with acne conglobate who do not respond to conventional treatment, as demonstrated by Yiu et al. (40) and Sand and Thomsen (13). According to multiple authors, the administration of TNF inhibitors, specifically Adalimumab, Etanercept, and Infliximab, has the potential to effectively treat various forms of inflammatory acne and other systemic inflammatory conditions, such as psoriasis (39, 40).

### 3.7. Adalimumab in acne pustolosa

Inflammatory bowel diseases, such as Crohn's disease or ulcerative colitis, are often associated with extraintestinal manifestations, such as inflammation of the skin tissue. Rispo et al. (41) presented a case report of ulcerative colitis in a 22-year-old male, who has worsened acne pustolosa, after treating with steroids and azathioprine. This negatively affects the patient's quality of life. It was then decided Adalimumab was then administered after which his condition of ulcerative colitis and acne pustolosa improved significantly (41).

## 4. Discussion

Acne, a common skin disorder, can be treated using a variety of options, such as topical medications, oral antibiotics, anti-androgens, or isotretinoin. However, a subset of patients may exhibit resistance or intolerance to these therapies. Cytokine levels, specifically TNF-α, IL-17, and IL-1β, have been found to increase in acne. As a result, the use of TNF inhibitors has been explored as a potential treatment for severe and refractory acne, as well as in the setting of inflammatory syndromes (42).

The patient in this study did not respond to traditional acne treatment, and his condition worsened, as was observed in the other case reports discussed in our literature review. Although photodynamic therapy is commonly used in the treatment of acne, to the best of our knowledge, there are no studies on the combined use of photodynamic therapy and adalimumab. We hypothesize that the combination of ALA-PDT and Adalimumab may offer several potential benefits for the management of acne vulgaris, including: (1) increased efficacy: combining two different treatment modalities with distinct mechanisms of action can enhance the overall effectiveness of treatment. ALA-PDT targets sebaceous glands and reduces sebum production, whereas adalimumab reduces inflammation by inhibiting TNF-α (33). Together, they can provide a more comprehensive treatment approach that targets multiple aspects of acne vulgaris (18). (2) Reduction in side effects: Adalimumab is associated with potential side effects such as injection site reactions, infections, and allergic reactions (43). The risk of adverse effects could potentially be lowered by using the ALA-PDT combination with Adalimumab, which may result in a reduction of the required Adalimumab dose (44). (3) Long-term remission: ALA-PDT has been shown to provide long-term remission of acne vulgaris, with some patients remaining acne clear up to 12 months after treatment. The combining of ALA-PDT with Adalimumab may enhance the duration of remission. (4) Improved quality of life: Acne vulgaris can have a significant impact on a person's quality of life, affecting self-esteem and confidence. By using a combination of ALA-PDT and Adalimumab, patients may experience a more rapid improvement in their acne symptoms, leading to a boost in self-confidence and an overall improvement in quality of life (17, 27, 45–48). Furthermore, despite the positive results demonstrated in case reports, no clinical trials have been conducted to examine the safety and efficacy of adalimumab (ADA) in treating acne. Our report suggests that the combination of ALA-PDT and adalimumab may be a safe and effective treatment option for moderate to severe acne vulgaris. Thus, there are significant knowledge gaps in this area, emphasizing the necessity for additional research on the use of ADA for acne treatment. This underscores the importance of conducting further studies to assess the potential benefits and risks associated with adalimumab therapy in individuals with acne.

## 5. Limitations

The use of TNF inhibitors, such as adalimumab, has been reported to offer partial or total improvement of acne with minimal side effects; however, in a minority of patients, it may actually cause the onset of acne, leading to the suspension of treatment. Although existing studies on the use of TNF inhibitors have only focused on male populations, it is crucial to consider their effectiveness and safety in female patients. Given the limited sample sizes in the literature, careful interpretation of the results is necessary, and further research is needed to determine the efficacy and safety of TNF inhibitors as a treatment for acne that does not respond to conventional treatments.

## Data availability statement

The original contributions presented in the study are included in the article/supplementary material, further inquiries can be directed to the corresponding author.

## Ethics statement

Ethical review and approval was not required for the study on human participants in accordance with the local legislation and institutional requirements. The patients/participants provided their written informed consent to participate in this study. Written informed consent was obtained from the individual(s) for the publication of any potentially identifiable images or data included in this article.

## Author contributions

All authors listed have made a substantial, direct, and intellectual contribution to the work and approved it for publication.
